# Identification of VIMP as a gene inhibiting cytokine production in human CD4+ effector T cells

**DOI:** 10.1016/j.isci.2025.113099

**Published:** 2025-07-24

**Authors:** Christophe M. Capelle, Ni Zeng, Egle Danileviciute, Sabrina Freitas Rodrigues, Markus Ollert, Rudi Balling, Feng Q. HeFeng

## Main text

(iScience *24*, 102289; April 23, 2021)

In the originally published article, author Feng Q. HeFeng’s name appeared as “Feng Q He.” This has now been updated to “Feng Q. HeFeng.”

